# Resource Partitioning in Food, Space and Time between Arctic Charr (*Salvelinus alpinus*), Brown Trout (*Salmo trutta*) and European Whitefish (*Coregonus lavaretus*) at the Southern Edge of Their Continuous Coexistence

**DOI:** 10.1371/journal.pone.0170582

**Published:** 2017-01-25

**Authors:** Hallvard Jensen, Mikko Kiljunen, Rune Knudsen, Per-Arne Amundsen

**Affiliations:** 1 Department of Natural Resources and Rural Development, Norwegian Institute of Bioeconomy Research, Tromsø, Norway; 2 Department of Biological and Environmental Sciences, University of Jyväskylä, Jyväskylä, Finland; 3 Department of Arctic and Marine Biology, UiT The Arctic University of Norway, Tromsø, Norway; National Taiwan University, TAIWAN

## Abstract

Arctic charr and European whitefish are considered to be strong competitors in lakes, with the latter usually being the superior species. However, high niche plasticity and lake morphometry may suggestively facilitate resource partitioning and coexistence between charr and whitefish. Here, we explore the trophic niche utilization (diet and habitat use) of charr and whitefish co-occurring with brown trout in the deep and oligotrophic Lake Fyresvatnet, southern Norway (59°05’N, 8°10’E). Using CPUE, stomach contents and stable isotope analyses, a distinct resource partitioning was revealed between brown trout and the other two species. Brown trout typically occupied the littoral zone, feeding on benthic invertebrates, surface insects and small-sized whitefish. In contrast, charr and whitefish were predominantly zooplanktivorous, but diverged somewhat in habitat utilization as charr shifted seasonally between the profundal and the littoral zone, whereas whitefish were found in the upper water layers (littoral and pelagic habitats). Accordingly, the stable isotope values of carbon (δ^13^C) reflected a pelagic orientated prey resource use for both charr and whitefish, whereas brown trout had elevated carbon and nitrogen (δ^15^N) signatures that reflected their benthivore and piscivore diet, respectively. The findings suggest that charr may not rely upon the profundal zone as a feeding habitat but as a refuge area, and may coexist with whitefish if a third competitive and predatory species like brown trout co-occur in the lake. The study indicates that a general high habitat plasticity of Arctic charr may be essential in the presently observed coexistence with a competitively superior fish species like whitefish, and that a third fish species like brown trout may facilitate this particular fish community structure.

## Introduction

Resource partitioning among species is an essential part of their ecology, and the fundamental niche of a species is rarely if ever fully utilized because the presence of other species and the local available ecological conditions will restrict their niche use. The effects of this narrow range of niche conditions define how resources could be/are partitioned [1,2], and may contribute to minimize the impacts of interspecific interactions and thereby facilitate the coexistence of species with relative similar ecology [3–5]. However, co-existence of two species with similar ecology may also be facilitated by the presence of a third competitive species, as suggested by the theoretical framework of Abrams and Rueffler [6].

In lakes, two frequently studied fish species concerning niche partitioning are Arctic charr (*Salvelinus alpinus*) and European whitefish (*Coregonus lavaretus*). The two species have similar fundamental trophic niches in terms of habitat and diet use, but with whitefish considered as the superior competitive species [7–9]. Compared to Arctic charr, whitefish are especially considered to be a superior zooplanktivorous predator [7]. While resource partitioning of the trophic niche of lacustrine fish communities frequently receives most attention, the dynamic mechanisms structuring the species composition may also be explained in terms of lake morphometry [10–11], or by the presence of a third competitive species that may moderate the effects of the competition (like e.g., grayling *Thymallus thymallus* [9], or perch *Perca fluviatilis*, [11].

Arctic charr is the World´s northernmost freshwater species, are typically cold-water adapted [12–13], and thereby seem to be vulnerable to extinction due to warmer climate especially at their southern edge of their distributions [14–16]. In warmer climate regions, cold-water adapted species like Arctic charr needs refuges in deep lakes, as their temperature preference is a trait that seems relatively fixed throughout their distribution [17–18]. Thus, they potentially will be outcompeted by species like whitefish and/or brown trout at their southern edge of distribution (e.g. [12,19]. The three species are considered to have opportunistic and potentially wide trophic niches (habitat and dietary use) with high phenotypic plasticity [9,10,20–22], and likely provide convenient and well-defined spatial and dietary entities for examining resource partitioning. In a small number of deep lakes of southern Fennoscandia, coexistence of Arctic charr and whitefish may occur when an extensive profundal zone offers a dietary and spatial niche refugee for the charr [7,11]. Here, we present results of their co-occurrence in a deep and oligotrophic lake (Lake Fyresvatn, Norway) from the southern edge of the continuous distribution of Arctic charr that potentially display new insight of niche partitioning and trophic plasticity of these three dominant lacustrine salmonids. A previous study in the lake revealed an ontogenetic shift from benthic invertebrates to small-sized whitefish for brown trout at sizes >30 cm [23].

Studies of the trophic niche of fishes have successfully used stable isotope analyses (SIA) of nitrogen (δ^15^N) and carbon (δ^13^C) to provide additional information to the traditional methods using stomach content data (SCA) and habitat preference [24,25]. The δ^13^C signatures provide information about the sources of carbon (benthic vs. pelagic production) at the base of the food web and thus the principal diet and/or habitat use of the consumers, whereas δ^15^N provides measures of the energy transfer and trophic positions of individuals in natural communities [26]. The advantage by using stable isotopes is that they reflect the diet integrated over a longer period of time, whereas SCA only reveals which prey that recently have been ingested, although with a better taxonomical resolution. Hence, SIA used in combination with SCA reveals both long and short-term aspects of the feeding ecology, providing an effective tool for studies of the trophic niche use, resource partitioning patterns in fish and energy transfer within a given food web [26–27].

The present study aims to explore the trophic niche (habitat and dietary use) of lacustrine Arctic charr at the southern edge of their continuous distribution range, co-occurring with specialized and superior competitors such as the zooplanktivore European whitefish and the benthivore brown trout in a deep oligotrophic lake. We used habitat utilization (CPUE values), stomach contents data and naturally occurring stable isotopes of nitrogen and carbon as short- and long-term time-integrated tracers of the trophic niche use of the three species. We expected that the co-existence of the three salmonids is related to species-specific trophic niche preferences. We firstly hypothesized that benthivore and piscivore brown trout occupy the littoral zone, whereas whitefish occupy the pelagic habitat feeding on zooplankton. Secondly, we hypothesized that Arctic charr may co-exist with the other species because of their general high niche plasticity to be able to better utilize the deep-water resources of a lake ecosystem.

## Materials and Methods

### Study site

Lake Fyresvatn (59°05’N, 8°10’E; 281.1 m.a.s.l.) is a deep (377 m) and oligotrophic lake (Table 1), situated in the Telemark county of southern Norway. The lake area is relatively large (49.6 km^2^), and part of the Arendal watercourse with a total catchment area of 4025 km^2^. The lake has served as a hydroelectric reservoir since 1973, with a maximum water regulation of 4.5 m, but the annual fluctuations are usually less than 2.5 m. Coniferous woodlands dominates the catchment area, and most of the shoreline and adjacent littoral zones consist of stony substrates. Regularly from 1997, the lake was limestone treated (8000 tons of dolomites) enhancing the pH from 5.48 to 6.01 in 2005 (i.e. prior to our field sampling). Peak temperature in July-August may reach 18–20°C, and the Secchi depth ranging from 8–10 m with Tot P and N of 5 μg·l^-1^ and 130 μg·l^-1^ respectively [28–29] (Table 1). The ice-free season in the lake lasts from March-April to November-December. The fish species in the lake are Arctic charr, whitefish and brown trout. All three species are native and are exploited in a commercial fishery sustaining a total yield of 8 − 10 tons annually with whitefish as the dominant resource.

**Table 1:** The physical and water chemistry (based on replicate water samples from 1 m depth) characteristics of Lake Fyresvatn.

Parameter	Value
Surface area (km^2^)	49.6
Max depth (m)	377
Mean depth (m)	30
Secchi depth (m)	8–12
Color (mg Pt·l^-1^)	6
pH	6.0
Alcalinity (μeq·l^-1^)	0.3
Ca (mg·l^-1^)	1.03
Tot P (μg·l^-1^)	5
Tot N (μg·l^-1^)	130

### Field sampling

**Fig 1:**
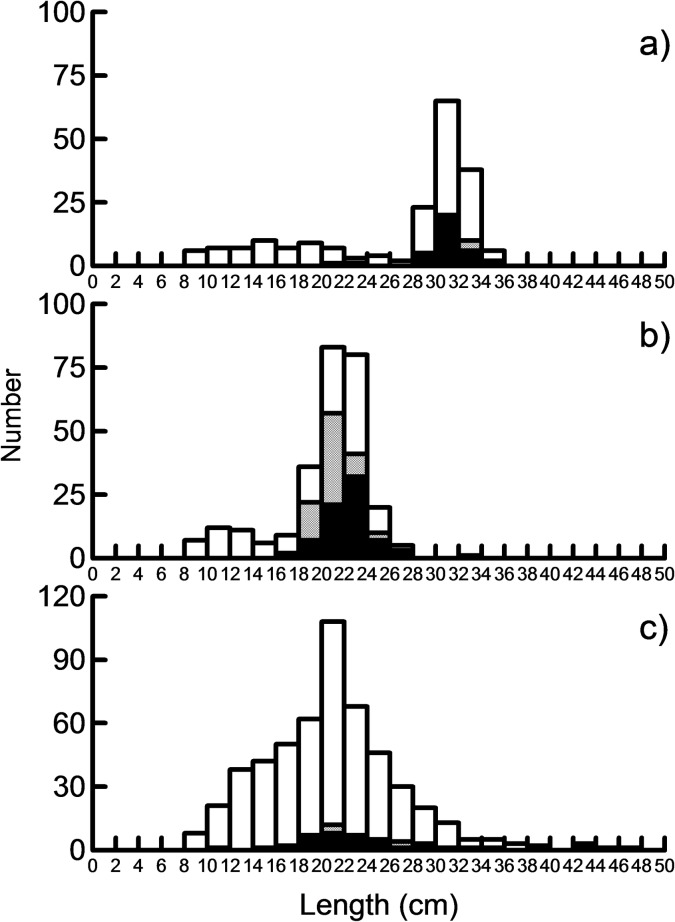
Length distribution of fish. Length distribution of a) whitefish, b) Arctic charr and c) trout in the survey gillnet catches in Lake Fyresvatn. Open bars show immature fish, and hatched and black bars matured males and 0females, respectively.

Water samples was taken in August 2005 as two replicate samples at 1 m depth offshore close to the pelagic gill nets, and frozen for later standard analyses (Fig 1). Fish were euthanized by means of cerebral concussion prior to sample collection. A fishing permission is required from the fishing right owner, which on Government land in Telemark county is the County Governor of Telemark with legal authority through LOV 1992-05-15 nr 47. § 13. Accordingly, we obtained permissions for the gill net fishing in Lake Fyresvatnet from the County Governor. No ethical permission is required from the Norwegian Animal Research Authority for collection with gill nets and the associated sacrifice of fish (FOR 1996-01-15 nr 23, the Norwegian Ministry of Agriculture and Food).

Fish were sampled during June, August and October in 2005 and 2006 with survey gill nets (knot to knot) of 10, 12.5, 15, 18·5, 22, 26, 35 and 45 mm in the littoral (0–15 m depth), profundal (> 15 m depth) and pelagic habitat (offshore, 0–6 m depth). Some additionally large-sized, piscivorous brown trout for SCA and SIA were sampled using single gill nets with mesh sized 35–52 mm (1.5 m deep and 30 m long) and trap nets (for SIA only) as a supplement in the littoral habitat zone.

Habitat use and density of fish were compared by estimating catch per unit effort (CPUE; number of fish caught per 100 m^2^ net area per night) of each species at the different sampling occasions. Each fish was measured for total length (L_T_; 0.1 cm), weighted (g), and the stomach was removed and stored in 96% ethanol for further stomach content analyses (SCA). For SIA, a small piece of muscle from the lateral muscle below the dorsal fin of each species were sampled (random subsamples, Arctic charr: n = 58; whitefish: n = 58 and brown trout: n = 170) and deep-frozen (< − 20°C).

For baseline and putative prey sources and isotopic signatures, pooled benthic invertebrate samples were collected during August using an Ekman dredge in profundal zone (n = 6), dip nets in the littoral zone (n = 6), whereas zooplankton samples (n = 6) was collected with a 100-μm mesh plankton net with a diameter of 30 cm hauled vertically in the pelagic habitat. For putative prey fish sources, muscle samples for SIA were in addition taken from a subsample of whitefish (n = 5, range: 13·2–16·9 cm *L*_T_), charr (n = 5, range: 11·5 − 15·5 cm *L*_T_), and brown trout (n = 5, range: 10·0–11·0 cm *L*_T_). The baseline and putative prey source samples were stored and deep-frozen before the subsequent SIA.

### Dietary analyses

The stomach contents of the fish were identified and categorized into a total of six different prey groups including (i) copepod zooplankton (Cyclopoida and Calanoida sp.), (ii) cladoceran zooplankton (*Bosmina* sp., *Daphnia* sp., *Holopedium* sp. *Bythotrephes longimanus*), (iii) benthic cladoceran (chydorids, *Eurycercus lamellatus*.), (iv) benthic invertebrates (*Ephemeroptera* sp., *Trichoptera* sp., *Plecoptera* sp., *Odonata* sp., *Coleoptera* sp. and *Chironomidae* spp.), (v) pleuston (emerging pupae and adults of aquatic and terrestrial insects), and (vi) fish.

The stomach fullness (in volume) was determined on a scale from 0 (empty) to 100% (full), and the fullness contribution of each diet category was estimated such that the sum of all categories equaled the total stomach fullness. The proportion of each diet category was expressed in percentage as prey abundance (%A_*i*_):

$$\%A_{i}=100\;x\;\sum F_{i}/\sum F_{t}$$

where *F*_*i*_ is fullness for diet category *i* and *F*_*t*_ is the total stomach fullness [30]. Proportion of empty stomachs was 29.9, 39.4 and 34.1 for brown trout, Arctic charr and whitefish, respectively.

The habitat and dietary overlap between Arctic charr, whitefish and brown trout were calculated using Schoener’s [31] index:

$$\alpha =1-0.5\left(\sum_{i=1}^{n}\left|P_{xi}-P_{yi}\right|\right)$$

where *P*_*xi*_ is the proportion of habitat/prey group *i* used by species *x*, *P*_*yi*_ the proportion of habitat/prey group *i* used by species *y*, and n the number habitat/prey categories. The index gives α-values from 0 to 1, where 0·00 and 1·00 indicates no overlap and complete overlap, respectively. The diet similarity is considered to be biological significant at an index value ≥ 0.60 [31]. Our findings revealed a similar pattern of diet and habitat use between sampling years (i.e. α > 60), and we therefore merged the samples (both SCA and SIA) from the two years to provide a larger and more reliable dataset.

PERMANOVA analysis was used to test the differences in diet composition between the three species, using the PRIMER v7 multivariate statistics package including the PERMANOVA+ add-on module [32]. The test used the Bray-Curtis similarity matrices constructed from the six different prey groups that had been generated from the prey abundance data (%A_*i*_), comparing the diet contribution among the three species (Arctic charr, brown trout and whitefish), habitat (littoral, profundal and pelagic) and season (June, August and October). In this test, species, habitat and season were fixed factors, whereas food resources were considered as dependent factors. All test were permutated 999 times under a reduced model [32].

### Stable isotope analyses

Fish and invertebrate samples for stable isotope analysis were freeze-dried to a constant weight. Samples were ground to fine powder using mortar and pestle and stored frozen in glass vials prior to analysis. Stable isotopes of carbon and nitrogen were analysed at the University of Jyväskylä, Finland, using a Carlo Erba Flash EA1112 elemental analyser connected to a Thermo Finnigan DELTA^plus^Advantage continuous flow stable isotope-ratio mass spectrometry (CF-IRMS). Results are expressed using the standard δ notation as parts per thousand (‰) difference from the international standards. The reference materials used were internal standards of known relation to the international standards of Vienna Pee Dee belemnite (for carbon) and atmospheric N2 (for nitrogen). Dried pike (*Esox lucius* L.) white muscle was used as an internal working standard and two replicate standards were run repeatedly after every ten samples in each sequence. Internal precision was always better than 0.2‰, based on the standard deviation of replicates of the standards. Sample analysis also yielded percent carbon and nitrogen (by weight) used in the models.

### Stable isotope models

Freeware package SIAR 4.2 [33], solving linear mixing models in the R (version 3.3.0) statistical computing environment, was used to determine the contribution of different prey to the diet of three fish species. Prey δ^15^N and δ^13^C values were corrected for trophic enrichment (Δ) using respective mean fractionation factors (± SD) of 3.23 ± 0.79‰ and 1.03 ± 0.29‰ [23]. Original, un-lipid-normalized data were used in the models. Therefore, concentration dependence, i.e. proportions of C and N in in dietary sources, was included in to the models [34,35]. In general, number of prey in the mixing models should be kept as low as possible, with only the most important sources included [36]. Following these tenets, the three most important prey groups were selected in to the models for each fish species. For trout average and standard deviation of δ^13^C and δ^15^N values from samples of the three mostly preferred groups of prey species in Lake Fyresvatnet (fish; whitefish (n = 5), charr (n = 5) and 10.0–11.0 cm juvenile trout (n = 5); benthic invertebrates (n = 12), and zooplankton (n = 6) were inserted into the model. Since gut content analysis did not show any piscivorous behavior for whitefish and charr, following three dietary sources were selected for these species; profundal invertebrates (n = 6), littoral invertebrates (n = 6) and zooplankton (n = 6). All the mixing model results are reported as mean of all feasible solutions with 5 − 95th percentiles of the distribution ranges.

To investigate niche width of three studied fish species, we estimated the community metric *total area* (TA) i.e. the minimum area where all the individuals of the particular species are bounded on in isotope bi-plot and standard ellipse area (SEAc) as a measure of the core population isotopic niche width [36]. Niche overlap was calculated based on SEAc ellipses and the extent of overlap ranging from 0 to 1, with values closer to 1 representing more overlap. Metric areas and their overlaps were calculated using a Stable Isotope Bayesian Ellipses in R (SIBER) package for R v.2.10.1. [37].

## Results

### Size distribution, habitat use and CPUE

The samples comprised 1080 fishes with brown trout as the dominant species (52.3%), followed by charr (32.8%) and whitefish (14.9%). From the survey catches, the length distribution of the fish displayed a wide size range of brown trout (range: 8.3–48.0 cm; mean 21.0 cm ± 6.6 SD), a more narrow size range of charr (range: 8.1–28.2 cm; mean 20.4 cm ± 4.0 SD) and an intermediate size-range of whitefish (range: 8–36 cm; mean 26.6 cm, ± 7.6 SD) (Fig 1).

**Fig 2:**
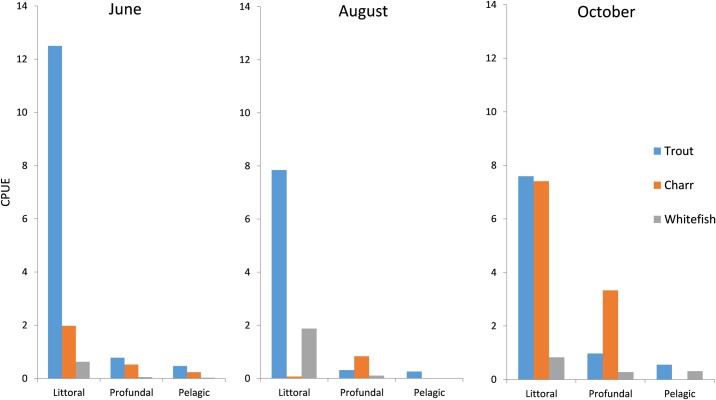
Fish density and habitat. Density (CPUE) of trout, Arctic charr and whitefish in the three principal (littoral, profundal and pelagic) habitats throughout the ice-free season in Lake Fyresvatn.Throughout the season, trout was predominately caught in the littoral catches, the CPUE being highest in June (12.3) and almost equally levelled in August (7.8) and October (7.5). Trout was present in the profundal and pelagic catches, but the density was low (CPUE: 0.1–1.0).

The habitat use of the fish was characterized by an overall dominance of trout in the littoral habitat, whitefish was commonly present in both the littoral and pelagic habitat, whereas charr dominated the catches in the profundal zone throughout the season (Fig 2). The highest CPUE (7.3) of Arctic charr was, however, observed in the littoral zone in October, but the species was in contrast almost absent in the August littoral catches (i.e. when water temperature was at the highest). Charr also appeared at low density in the pelagic catches in June (CPUE: 0.3).

Whitefish was regularly present in the littoral zone throughout the season, having a peak density in August and an overall higher CPUE compare to charr. The density of whitefish in the profundal and pelagic were consistently low (CPUE: 0.1–0.5). Whereas the overall CPUE in terms of number of fish was lowest for whitefish (15%) among the three salmonids, their total contribution in all habitats constituted 29% of the total biomass (cf. Fig 1).

### Diet

#### SCA–Littoral

**Fig 3:**
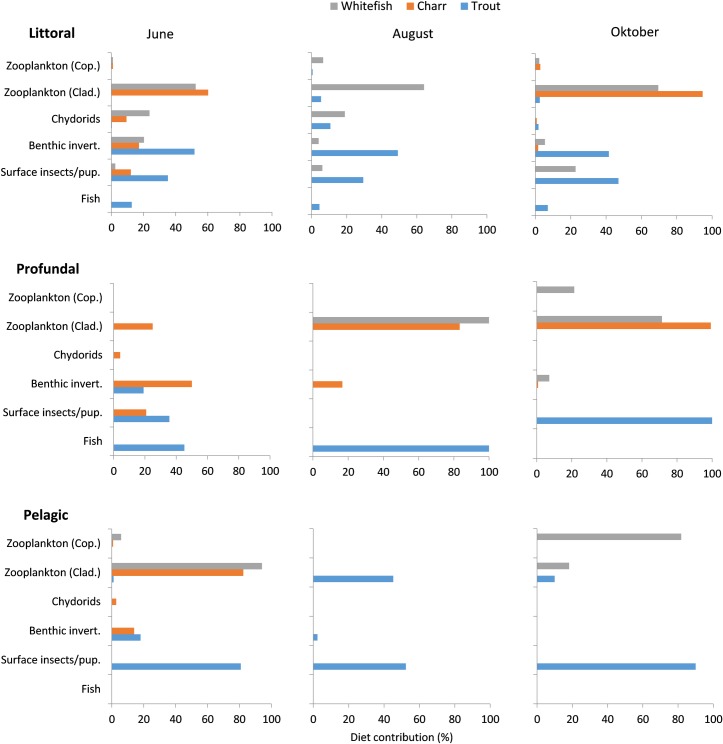
Diet contribution. Seasonal diet contribution ((%A_*i*_) of main prey groups in the stomach contents of whitefish, Arctic charr and trout caught in littoral, profundal and pelagic habitats of Lake Fyresvatn.

Charr had a mixed diet in June with a dominance of zooplankton (62% cladocerans), and a lower (~20%) proportion of either benthic invertebrates, chydorids or surface insects/pupae. Charr caught in October fed heavily on cladoceran zooplankton (mainly *H*. *gibberum* and *B*. *longispina*). In contrast, brown trout caught in the littoral zone had mainly eaten benthic invertebrates and surface insects independent of sampling period (Fig 3). Larger trout (>30 cm) fed almost exclusively on fish, predominantly small-sized whitefish. For whitefish, cladoceran zooplankton consistently dominated the diet throughout the season (53–70%), whereas the proportion of chydorids and benthic invertebrates decreases towards autumn when surface insects/pupae became more important.

#### Profundal and pelagic

Charr caught in the profundal had a mixed diet with a dominance of benthic invertebrates in June, but shifted towards feeding on cladoceran zooplankton in August and September. Charr caught in the pelagic fed heavily on cladoceran, and to a minor extent (<10%) on benthic invertebrates. The few trout caught in the profundal habitat had a mixed diet of fish, benthic invertebrates, and surface insects/pupae in June, but shifted towards exclusively piscivory or insects/pupae in August and October, respectively. Trout were infrequent caught in the pelagic zone and had mainly eaten surface insects/pupae throughout the season, with an important contribution of cladoceran zooplankton in August. Among the small number of whitefish caught in the profundal zone, the diet was dominated by cladoceran zooplankton. Whitefish caught in the pelagic fed heavily on zooplankton, with cladoceran and copepods dominating in August (94%) and October (82%), respectively (Fig 3).

### Interspecific dietary relationships

The diet composition differed significantly among the three species (PERMANOVA; P = 0.001) and among habitat (P = 0.012), but not by season (P = 0.308) (Table 2). With regards to other two-way interactions between species and habitat, species and season or habitat and season, the combined effects was generally low between the three salmonids (Table 2).

**Table 2:** Degrees of freedom (d.f.), Mean squares (MS), pseudo-*F* ratios and significance levels (*P*) for a series of permutational analysis of variance (PERMANOVA) tests, using Bray-Curtis similarities matrices derived from the mean percentage volumetric contributions (%A_i_) of the main dietary categories (n = 6) by species ˟ habitat ˟ season for Arctic charr, brown trout and whitefish in Lake Fyresvatn.

	d.f.	MS	Pseudo-*F*	*P*(perm)	Uniq perms*
**Main effects**					
Species	2	10553	8,19	**0,001**	999
Habitat	2	4378	3,40	**0,012**	999
Season	2	1713	1,33	0,308	998
**Interactions**					
Species ˟ habitat	4	1834	1,42	0,222	999
Species ˟ season	4	1718	1,33	2,256	999
Habitat ˟ season	4	1614	1,25	0,351	999
Residual	8	1288			
Total	26				

*Uniq perms; permutations performed. Significant values shown in bold.

In the littoral habitat, charr and whitefish showed regularly high dietary overlap (Schoener’s α 72–83%), usually related to a common utilization of cladoceran zooplankton (Table 3, cf. Fig 3). Between trout and the two other species, however, the overlap was generally low and scored from 5–31%, indicating a distinct diet partitioning. In the profundal and pelagic, a similar patterns occurred between whitefish and charr, the dietary overlap being highest in the profundal in August and in the pelagic in June. For brown trout, the highest diet overlap (40%) occurred in the profundal zone when co-occurring with charr, but in general the overlap was low (>15%) relative to both charr and whitefish.

**Table 3:** Dietary overlap (Schoener’s α) between Arctic charr, brown trout and whitefish caught in different lake habitats (littoral, profundal and pelagic) through the ice-free season in Lake Fyresvatn. Significant values shown in bold.

Habitat	Species	June	August	October
**Littoral**	Charr vs whitefish	**83**	-	**73**
	Charr vs trout	30	-	5
	Trout vs whitefish	23	27	31
**Profundal**	Charr vs whitefish	-	**83**	**72**
	Charr vs trout	40	0	0
	Trout vs whitefish	-	0	0
**Pelagic**	Charr vs whitefish	**83**	-	-
	Charr vs trout	15	-	-
	Trout vs whitefish	1	-	10

### Stable isotope analyses

#### Bi-plot signatures

**Fig 4:**
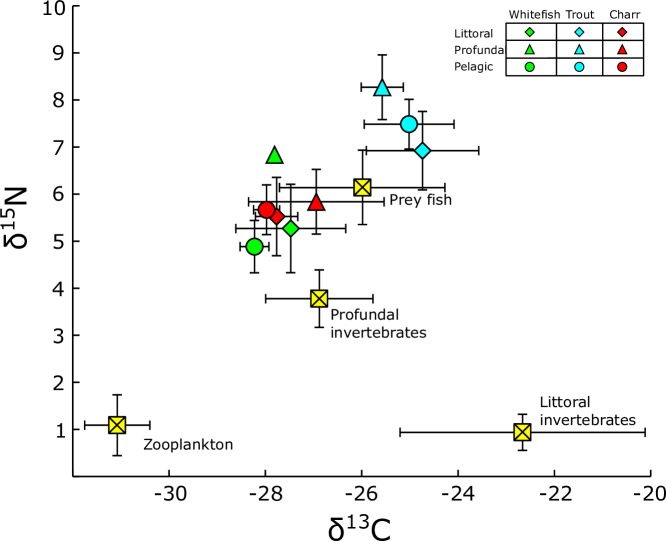
Bi-plot signatures of δ^15^N and δ^13^C stable isotopes. Average (±SD) stable isotope values of the three fish species caught from different habitats in Lake Fyresvatn and their main dietary sources. Trout diet consists of fish (whitefish, charr and juvenile trout), benthic invertebrates and zooplankton. Diet of whitefish and charr consisted of profundal invertebrates, littoral invertebrates and zooplankton.

Arctic charr and whitefish showed rather similar isotope values (Fig 4), but brown trout had clearly more enriched δ^15^N and δ^13^C values compared to other two species (Kruskal-Wallis test, P < 0.001). No clear differences in the isotope values were found between fish caught from the different habitats, although fish caught from profundal seemed to have slightly higher δ^15^N values compared to those sampled from other habitat types. The major food sources were well separated in the stable isotope space, despite large variation in the littoral invertebrate signatures (Fig 4).

#### SIAR mixing models and SIBER community metrics

**Fig 5:**
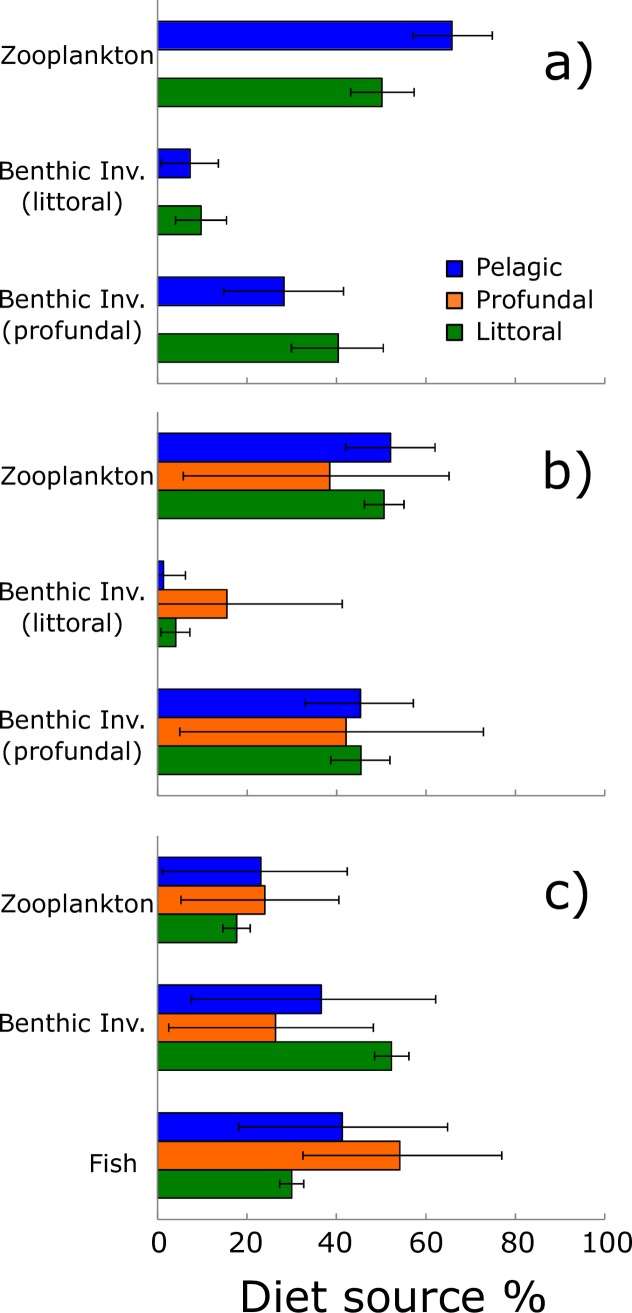
SIAR model estimates and diet sources. SIAR model percent mode estimates and accompanying high and low 95% credibility intervals for potential diet sources of a) whitefish, b) Arctic charr and c) trout from Lake Fyresvatn caught in the three principal (littoral, profundal and pelagic) habitats.

For Arctic charr and whitefish, the littoral food sources represent only small proportion of their overall diet (Fig 5). Arctic charr rely almost equally on both zooplankton and profundal prey sources, whereas whitefish diet heavily rely on zooplankton sources, although benthic invertebrates from profundal zone were also present. No large differences were found in the diet proportions of Arctic harr and whitefish caught in different habitats, although there was slight tendency towards pelagic caught fish to feed more on zooplankton. For brown trout, these differences were more pronounced and fish caught from pelagic and profundal were more piscivorous than those caught form littoral zone, which in contrast seemed to rely mostly on benthic invertebrates.

**Fig 6:**
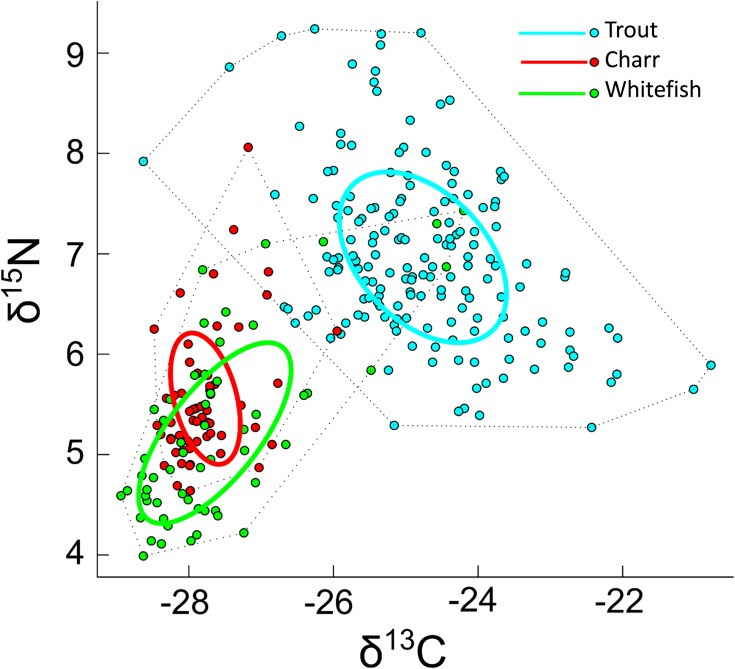
Dietary isotopic niches. Dietary isotopic niches of the three species of fish in Lake Fyresvatn. Dots represent each individual fish in δ^15^N and δ^13^C space, ellipses are standard core areas (SEAc) of each species and dotted lines are estimated community metric total areas (TA).

Calculated SIBER metrics, core niche (SEAc) and total area (TA), support the findings from gut content analysis and SIAR mixing models. Trout obtained clearly wider dietary niche (SEAc = 2.81 and TA = 18.13) compared to whitefish (SEAc = 2.18 and TA = 8.21) and charr (SEAc = 0.93 and TA = 5.14) (Fig 6). Strong overlap between charr and whitefish core niche ellipse areas (SEAc overlap = 0.61) was observed, whereas trout niche was totally separated from other two (SEAc overlap = 0), suggesting strong niche partitioning in trout (Fig 6).

## Discussion

We found a distinct stable resource partitioning between Arctic charr and whitefish co-occurring with brown trout, reflected by their temporal and spatial trophic niche utilization. At the habitat dimension, the three salmonids behaved as hypothesized. The benthivore brown trout was the dominant species in the littoral zone, as commonly observed throughout their distribution range [25, 38–39]. The zooplanktivore whitefish resided mainly in the preferred upper water layers (i.e. the littoral and pelagic habitats) which corresponds well to other studies [11,40]. In contrast, Arctic charr seemed to switch between the profundal and the littoral zone of the lake and more or less avoided the pelagic zone. In the present study, the zooplanktivore Arctic charr used the profundal zone more as a refuge habitat although they may frequently utilize the profundal zone also as a feeding habitat [41], especially under competitive interactions with other species [7]. Although Arctic charr and whitefish was partial segregated with respect to habitat use, their dietary overlap was substantial with zooplankton as the main feeding resource according to both stomach contents and the stabile isotope signatures. The high contribution of zooplankton as prey for Arctic charr living in sympatry with whitefish was highly surprising as whitefish is considered as the competitive superior zooplanktivore of the two species [7]. However, Arctic char are found to exhibited higher growth efficiency (per unit of food) and appear to have out-competed for example brown trout from cold, low-productivity lakes [17,42], and this might partly be the explanation in the observed sympatric behavior with whitefish in our study. Arctic charr also showed a strong temporal and spatial variability in habitat preference with a peak littoral presence in October and an almost absence in this habitat in August. The fact that the charr were not exclusively displaced into the profundal zone and temporally utilized both zooplankton and benthic invertebrates despite the quite substantial deep-water areas in the lake and the presence of superior competitors in the more shallow habitats, illustrate their high niche plasticity and opportunistic behavior [10,21,40–43].

Based on SCA, trout and whitefish utilized mainly food resources (benthivore and zooplanktivore, respectively) from the lake habitat (littoral and pelagic zone, respectively) they where caught, but the zooplanktivore charr were infrequent caught in the pelagic habitat. On the other hand, the SIAR model indicate that the diet preferences of each fish species is not strongly correlated with the habitat where the fish was caught. These opposing observations are most likely a result of methodological differences as SCA commonly reflects the prey recently ingested, whereas stable isotopes display the diet integrated over a longer period of time [26]. Another explanation is the relative low number of stable isotope samples analysed per month that affect the temporal aspect of the stable isotope data and the robustness of the SIAR models. Nevertheless, as the fish species in the present study are highly mobile predators and capable of utilizing all lake habitats, the SIAR models suggests no clear habitat specific dietary segregation existing for one specific fish species at longer time scales. However, based on SCA and the CPUE results a clear trophic segregation is evident in a short-time scale and SCA provided a higher resolution in taxonomic composition of the diet compared to stable isotope methods. Numerous other studies have demonstrated that by using multiple methods to estimate diet composition improve the reliability of the results (e.g. [23,44–45]). Our results clearly illustrate that combining time-integrative methods (stable isotopes) and dietary snapshots (SCA) is particularly important in trophic plastic and opportunistic species in order to understand species interaction at varying temporal and spatial scales.

Our study demonstrated a classic niche segregation between Arctic charr and brown trout [37, 46–48] both from the habitat, dietary and stable isotope analyses. In sympatry with Arctic charr, the more aggressive brown trout typically displace the charr from the littoral to the inferior pelagic and/or profundal niches [25,37,49]. In sub-Arctic areas, Arctic charr seem to have a stable resource utilization in co-occurrence with brown trout, exploiting both the littoral and pelagic habitats and feeding substantially on benthic prey throughout ontogeny and by time [25,50–51]. In contrast, the zooplanktivore Arctic charr from the present study at the southern edge of their continuous distribution likely avoid upper water layers of the littoral and pelagic habitats during the warm period in August. This is most likely because the water temperature (18–19°C) is considerable higher than their general preference temperature around 12°C [17,52]. A deep and cold-water refuge as the profundal habitat offered in Lake Fyresvatn, therefore seems as an essential contribution for the charr population during hot summer periods. Zooplanktivore whitefish, on the other hand, mainly occupy the upper water layers in Fyresvatn. Our results suggest that the Arctic charr and whitefish have almost identical trophic niches from dietary and stable isotope signatures, which is quite uncommon compared to similar partitioning studies elsewhere [11,53]. Consequently, the two species seem to segregate along another niche dimension (i.e. the temperature gradient) during hot periods. However, during the other study months with lower water temperature, their trophic niche preferences were more similar. From both experimental and field studies, Arctic charr has shown to feed and grow during much lower temperature regimes than whitefish and brown trout [21,52,54]. Thus, at the southern edge of the continuous distribution of Arctic charr they may experience heavy resource competition with e.g., trout and whitefish (or other species), which combined with unfavorable abiotic environments may result in a relegation to deeper habitats. Our findings therefore suggest that ecological interactions and abiotic factors in concert may truncate the distribution of Arctic charr. Hence, a changing climate and increased resource competition may potentially lead to an eradication of these Arctic charr populations located at the southern edge of their continuous distribution (e.g. [14–16]).

Arctic charr may turn into piscivory when co-occurring with whitefish, but this is likely to occur more frequently in subarctic areas or in more species rich communities [55–58]. However, the piscivore niche in Lake Fyresvatn is totally occupied by the more aggressive brown trout [23], where large-sized specimens (>30 cm) was predominantly foraging on small-sized whitefish and to a minor extent on Arctic charr. This implicates that the presence of piscivore brown trout may have a negative impact on whitefish abundance through elevated predation pressure. Hence, even though the similar zooplanktivore niches with high diet overlap between Arctic charr and whitefish suggest a competitive impact on charr due to the presence of whitefish as found from other studies [7], the presence of the benthivore and piscivore brown trout may facilitate the coexistence of the two other species. This may occur as Arctic charr use the profundal habitat as a refuge area (e.g. away from predatory brown trout and superior zooplanktivore whitefish) but not as a feeding habitat. Thereby the Arctic charr have a stable diet utilization (zooplanktivory) but show plasticity in their habitat use. This co-existence of the three salmonids in Lake Fyresvatn have similarities to the findings from another system with three potentially competing salmonids species; Arctic charr, whitefish and grayling, *Thymallus thymallus* [9]. Amundsen et al. [9] considered their findings in the light of a model study of Abrams and Rueffler [6], addressing the ability of three consumer species to coexist along a one-dimensional resource axis where the presence of one species (e.g. grayling or brown trout) in a lake may contribute to facilitate the coexistence of two other species (e.g. Arctic charr and whitefish). The model suggests that coexistence of three species most likely may occur when the intermediate species (e.g. Arctic charr) is closer to one of the two other species (e.g. whitefish). The outcome of the model from Abrams and Rueffler [6] may very well apply to the situation in Lake Fyresvatn given the similarity in resource utilization between Arctic charr and whitefish, but more work are needed to confirm the potential facilitating role of brown trout in the observed coexistence of Arctic charr and whitefish. Future studies might extend the present work to investigate niche plasticity and resource partitioning among food webs, including observation from lower diversity systems to more complex salmonid lakes.

In conclusion, our study demonstrates co-existence and partial resource partitioning between three closely related salmonids. The co-existence of all three species is apparently related to the observed high spatial and dietary plasticity of the Arctic charr in general [10,11]. The observed trophic niche use of Arctic charr could be restricted due to high competition for zooplankton resources from whitefish in the pelagic zone, high predation risk from large-sized brown trout in the littoral zone and unfavorable abiotic conditions (i.e. warm water). The findings indicate that Arctic charr does not solely rely upon the profundal zone as an available feeding habitat but more as a refuge habitat, and thereby may coexist with whitefish if a third competitive and predatory species like brown trout co-occur in the lake. Thus, a niche plasticity in habitat utilization of Arctic charr appears essential in the observed coexistence with zooplanktivore whitefish and benthivore brown trout.

## References

[1] Schoener TW. Nonsynchronous spatial overlap of lizards in patchy habitat. Ecology 1970;51: 408–418.

[2] Pianka E.R. 2000. Evolutionary Ecology. Sixth Edition Benjamin-Cummings, Addison-Wesley-Longman: San Francisco; 2000.

[3] Gause GF. The struggle for existence New York: Hafner; 1934. 10.1126/science.79.2036.16-a 17821472

[4] Bøhn T, Amundsen PA, Sparrow A. Competitive exclusion after invasion? Biol Invasions 2008;10: 359–368.

[5] Jonsson B, Jonsson N, Hindar K, Northcote TG, Engen S. Asymmetric competition drives lake use of coexisting salmonids. Oecologia 2008;157: 553–560. 10.1007/s00442-008-1103-1 18629544

[6] Abrams PA, Rueffler C. Coexistence and limiting similarity of consumer species competing for linear array of resources. Ecology 2009;90: 812–822. 19341150 10.1890/08-0446.1

[7] Svärdson G. Interspecific population dominance in fish communities of Scandinavian lakes. Report of the Institute of Freshwater Research Drottningholm 1976;55: 144–171.

[8] Museth J, Borgstrøm R, Hame T, Holen LÅ. Predation by brown trout: a major mortality factor for sexually mature European minnows. J. Fish Biol. 2003; 62: 692–705.

[9] Amundsen PA, Knudsen R, Bryhni HT. Niche use and resource partitioning of Arctic charr, European whitefish and grayling in a subarctic lake. Hydrobiologia 2010;650: 3–14.

[10] Eloranta A.P., Kahilainen K.K., Amundsen P-A., Knudsen R., Harrod C. & Jones R.I. 2015. Lake size and fish diversity determine resource use and trophic position of a top predator in high-latitude lakes. Ecol. Evol. 2015; 5: 1664–1675. 10.1002/ece3.1464 25937909 PMC4409414

[11] Sandlund OT, Museth J, Næsje TF, Rognerud S, Saksgård R, Hesthagen T, et al Habitat use and diet of sympatric Arctic charr (*Salvelinus alpinus*) and whitefish (*Coregonus lavaretus*) in five lakes in southern Norway: not only interspecific population dominance? Hydrobiologia 2010;650: 27–41.

[12] Gerdeaux D. Does global warming threaten the dynamic of Arctic charr in Lake Geneva? Hydrobiologia 2011;660: 69–78.

[13] Elliott JM, Elliott JA. Temperature requirements of Atlantic salmon *Salmo salar*, brown trout *Salmo trutta* and Arctic charr *Salvelinus alpinus*: predicting the effects of climate change. J. Fish Biol. 2010;77: 1793–1817. 10.1111/j.1095-8649.2010.02762.x 21078091

[14] Winfield IJ, Fletcher JM, James JB. An overview of fish species introductions to the English Lake District, UK, an area of outstanding conservation and fisheries importance. J. Appl. Ichthyol. 2010; 26: 60–65.

[15] Jeppesen E, Mehner T, Winfield IJ, Kangur K, Sarvala J, Gerdeaux D, et al Impacts of climate warming on the long-term dynamics of key fish species in 24 European lakes. Hydrobiologia 2012;694: 1–39.

[16] Murdoch A, Power M. Assessing the food web impacts of an anadromous Arctic charr introduction to a sub-Arctic watershed using stable isotopes. Fisheries Management and Ecology 2013;20: 302–314.

[17] Larsson S. Thermal preference of Arctic charr, *Salvelinus alpinus*, and brown trout, *Salmo trutta* –implications for their niche segregation. Env. Biol. Fish. 2005;73: 89–96.

[18] Siikavuopio SI, Sæther BS, Johnsen H, Evensen T, Knudsen R. Temperature preferences of juvenile Arctic charr originating from different thermal environments. Aquat. Biol. 2014;48: 313–320.

[19] Graham CT, Harrod C. Implication of climate change for the fishes of the British Isles. J Fish Biol 2009;74: 1143–1205. 10.1111/j.1095-8649.2009.02180.x 20735625

[20] Sandlund OT, Næsje TF. Komplekse, laksefiskdominerte fiskesamfunn på Østlandet. 2st ed. In Borgstrøm R, Hansen LP, editors. Fisk i ferskvann. Landbruksforlaget: Oslo; 2000. pp. 109–130. (In Norwegian).

[21] Klemetsen A, Amundsen P-A, Dempson B, Jonsson B, Jonsson N, O’Connell MF, Mortensen E. Atlantic salmon *Salmo salar* L., brown trout *Salmo trutta* L. and Arctic charr *Salvelinus alpinus* (L.): a review of aspects of their life histories. Ecol Freshw Fish 2003a;12: 1–59.

[22] Jensen H, Kahilainen K, Amundsen P-A, Gjelland KØ, Tuomaala A, Malinen T, et al Predation by brown trout (*Salmo trutta*) along a diversifying prey community gradient. Can. J. Fish. Aquat. Sci. 2008;65: 1831–1841.

[23] Jensen H, Kiljunen M, Amundsen P-A. Dietary ontogeny and niche shift to piscivory in lacustrine brown trout Salmo trutta revealed by stomach content and stable isotope analyses. J Fish Biol. 2012;80: 2448–2662. 10.1111/j.1095-8649.2012.03294.x 22650427

[24] Fry B. Coupled N, C and stable isotope measurements using a dual-column gas chromatography system. Rapid Commun Mass Spectrom 2006;5: 750–756.

[25] Eloranta A, Knudsen R, Amundsen PA. Niche segregation of coexisting Arctic charr (*Salvelinus alpinus*) and brown trout (*Salmo trutta*) constrains food web coupling in subarctic lakes. Freshw Biol 2013;58: 207–221.

[26] Post DM 2002. Using stable isotopes to estimate trophic position: models, methods, and assumptions. Ecology 2002;83: 703–718.

[27] Vander Zanden MJ, Rasmussen JB. Primary consumer δ^13^C and δ^15^N and the trophic position of aquatic consumers. Ecology 1999;80: 1395–1404.

[28] Hindar A 2006. Arendalsvassdraget. In: Hindar A, editor. Kalking i vann og vassdrag − effektkontroll av større prosjekter 2005. Trondheim: Norway; 2006, pp. 2–30 (In Norwegian).

[29] Jensen H. Ecological factors affecting piscivory of brown trout (Salmo trutta L.) in northern lakes. Ph.D. Thesis, University of Tromsø. 2009.

[30] Amundsen PA, Gabler HM, Staldvik FJ. A new approach to graphical analysis of feeding strategy from stomach contents data–a modification of the Costello (1990) method. J Fish Biol 1990;48: 607–614.

[31] Wallace RK. An assessment of diet–overlap indexes. T. Am. Fish. Soc. 1981;110: 72–76.

[32] Clarke KR, Gorley RN. Getting started with PRIMER v7. PRIMER-E: 2015, Plymoth; 296pp.

[33] Inger R, Jackson AL, Parnell, A, Bearhop S 2010. SIAR V4 (Stable Isotope Analysis in R). An Ecologist’s Guide. Available: http://www.tcd.ie/Zoology/research/research/theoretical/siar/SIAR_For_Ecologists.pdf

[34] Phillips DL, Koch PL. Incorporating concentration dependence in stable isotope mixing models. Oecologia 2002;130: 114–125. 28547016 10.1007/s004420100786

[35] Kiljunen M, Grey J, Sinisalo T, Harrod C, Immonen H, Jones RI. A revised model for lipid-normalizing δ^13^C values from aquatic organisms, with implications for isotope mixing models. Journal of Applied Ecology 2006;43, 1213–1222.

[36] Fry B. Alternative approaches for solving underdetermined isotope mixing problems. Mar Ecol Prog Ser 2013;472: 1–13.

[37] Jackson AL, Inger R, Parnell AC, Bearhop S. Comparison isotopic niche widths among and within communities: SIEBER–Stable Isotope Bayesian Ellipses in R. J. Anim. Ecol. 2011;80: 595–602. 10.1111/j.1365-2656.2011.01806.x 21401589

[38] Langeland A, L`Abée-Lund JH, Jonsson B, Jonsson N. Resource portioning and niche shift in Arctic charr *Salvelinus alpinus* and brown trout *Salmo trutta*. J. Anim. Ecol. 1991; 60: 895–912.

[39] Kahilainen K, Lehtonen H. Resource use of native and stocked brown trout *Salmo trutta* L., in a subarctic lake. Fish Manag Ecol 2001;8, 83–94.

[40] Eloranta A, Siwertsson A, Knudsen R, Amundsen PA. Dietary plasticity of Arctic charr (*Salvelinus alpinus*) facilitates coexistence with superior competitively whitefish (*Coregonus lavaretus*). Ecology of Freshwater Fish 2011;20: 558–568.

[41] Knudsen R, Klemetsen A, Amundsen P-A, Hermansen B. Incipient speciation through niche expansion: an example from the Arctic charr in a subarctic lake. Proceeding of the Royal Society B;2006;273, 2291–2298. 10.1098/rspb.2006.3582 PMC1636095 16928630

[42] Finstad AG, Forseth T, Jonsson B, Bellier E, Hesthagen T, Jensen AJ, et al Competitive exclusion along climate gradients: energy efficiency influences the distribution of two salmonid fishes. Glob. Chang. Biol. 2011;4: 1703–1711.

[43] Corrigan LJ, Winfield IJ, Hoelzel AR, Lucas MC. Dietary plasticity in Arctic charr (*Salvelinus alpinus*) in response to long-term environmental change. Ecol Freshw Fish 2011;20: 5–13.

[44] Lehtiniemi M, Kiljunen M, Jones RI. Winter food utilization by sympatric mysids in the Baltic Sea, studied by combined gut content and stable isotope analyses. Mar. Biol. 2009;156: 619–628.

[45] Polito M.J., Trivelpiece W.Z., Karnovsky NJ, Ng E, Patterson WP, Emslie SD. Integrating stomach content and stable isotope analyses to quantify the diets of *Pygoscelid Penguins*. PLoS ONE 2011;6 e26642 10.1371/journal.pone.0026642 22053199 PMC3203888

[46] Nilsson NA. Interactions between trout and char in Scandinavia. T. Am. Fish. Soc. 1963;92: 276–285.

[47] Nilsson NA. Interactive segregation between fish species, In: Gerking S.D. (Ed.) The biological basis of fish production. Blackwell Scientific Publication; Oxford: 1967 pp. 295–313.

[48] Wootton RJ. Ecology of Teleost Fishes. 2st ed Dordrecht: Kluwer Academic Publications; 1998.

[49] Jansen PA, Slettvold H, Finstad AG, Langeland A. Niche segregation between Arctic charr (*Salvelinus alpinu*s) and brown trout (*Salmo trutta*): an experimental study of mechanism. Can. J. Fish. Aquat. Sci. 2002;59: 6–11.

[50] Persson L., Amundsen P.-A., De Roos A.M., Klemetsen A., Knudsen R. Primicerio, R. 2007. Culling prey promotes predator recovery—alternative states in a whole-lake experiment. Science 2007;316: 1743–1746. 10.1126/science.1141412 17588929

[51] Knudsen R, Primicerio R, Amundsen P-A, Klemetsen A. Temporal stability of individual feeding specialization may promote speciation. J. Anim. Ecol. 2010;79: 161–168. 10.1111/j.1365-2656.2009.01625.x 19796292

[52] Siikavuopio SI, Knudsen R, Amundsen PA. Comparative growth study of Arctic charr and European whitefish at low temperatures. Hydrobiologia 2010;650: 255–263.

[53] Museth J, Sandlund OT, Borgstrøm R. Coexistence between introduced whitefish (Coregonus lavaretus) and native Arctic charr (Salvelinus alpinus) depends on heavy whitefish exploitation. Adv. Limnol. 2007;60: 343–350.

[54] Amundsen PA, Knudsen R. Winter ecology of Arctic charr (*Salvelinus alpinus*) and brown trout (*Salmo trutta*) in a subarctic lake. Aqua Ecol. 2009;43: 765–775.

[55] L’Abée-Lund JH, Langeland A, Sægrov H. Piscivory by brown trout *Salmo trutta* L. and Arctic charr *Salvelinus alpinus* (L.) in Norwegian lakes. J. Fish Biol. 1992;41: 91–101.

[56] Amundsen PA. Piscivory and cannibalism in Arctic charr. J Fish Biol 1994;45: 181–189.

[57] Kahilainen K, Lehtonen H. Piscivory and prey selection of four predator species in a whitefish dominated subarctic lake. J. Fish Biol. 2003;63, 659–672.

[58] Eloranta A.P., Kahilainen K.K., Amundsen P-A., Knudsen R., Harrod C. & Jones R.I. 2015. Lake size and fish diversity determine resource use and trophic position of a top predator in high-latitude lakes. Ecol. Evol. 2015; 5: 1664–1675. 10.1002/ece3.1464 25937909 PMC4409414

